# Dental Caries and the Remedy

**Published:** 1876-07

**Authors:** Geo. Paine


### DENTAL CARIES AND THE REMEDY.

BY DR. GEO. PAINE.

*Read at a Meeting of the Mad River Dental Society, April, 1876, at Dayton, O.*

It is a notorious fact that must be patent to every one, either from painful experience or observation, that human teeth are greatly and generally predisposed to decay. That while some fortunate mortals are exempt from disease of the dental organs, as they are also from physical ailments, because blessed by the sound heritage of a sound constitution and subsequent good health, are heirlooms transmitted to them from a healthy ancestry, "who did not eat of the sour grapes that their children's teeth might be set on edge." Nevertheless a proportion of our population are cursed by the heritage of bad health and bad teeth, in all stages of decay and dental dilapidation, from slight or incipient caries, to the indiscriminate and ruthless disintegration of all the dental organs, reeking with disease and consequent putrefaction, rendering the patient an object of disgust, because of the foul effluvia emanating from his mouth, poisoning his whole system, rendering him a fit prey to the malignant and often fatal diseases, thus abridging his days and sending him to a premature grave. A telling and significant commentary on the sinfulness of disobedience to the physical laws which govern our being, teaching that when we sin we must and will suffer. "*Heredity* is defined to be that Biological Law, by which all living beings tend to repeat themselves." (*Ribot.*) In their progeny we know that this law obtains in all those diseases termed hereditary, such as scrofula, syphilis, rheumatism, etc., and we also know that from actual observation, that this biological law finds complete illustration and emphasis in respect to the teeth, anatomically, physiologically and pathologically. It is a fact admitting of no dispute, that if the teeth of parents possess any peculiarity of mal-formation or mal-position, or are carious in any peculiar way, this idiosyncrasy will be developed, to a greater or less extent, in the dentures of their children. This law asserts its potency in the configuration of the jaws, in the type and shade of the teeth, and in the character, quantity and location of the decay. With this hereditary tendency on the part of the teeth to decay, it is not surprising that when they are exposed to the corrosive action of acids, those chief factors of their destruction, they should so speedily disintegrate and become necessary subjects for dental manipulation, either for reparation or destruction. Having assumed that hereditary influence is a potent element, ready for action, whenever a developing cause is present in the shape of acid, existing in the saliva, or coming in contact with the teeth as a therapeutic agent, it may not be impertinent to inquire what can or should be done to avert, annul or modify this hereditary proclivity to dental disease. This enemy must be met, if possible, on the threshold and held in abeyance by hygienic means, such as correct habits of life and living; suitable clothing, judicious diet, pure air, plenty of sunlight, frequent bathing and an abundance of sound sleep. In a word we must adopt such a diet as will feed and nourish the starved and starving teeth, such a regimen as will foster and build up and make the "*mens sana in corpore sano.*" It is an admitted fact that there are articles of food which supply the elements or proximate principles which these starved teeth require, rendering them strong and flint like in texture, thus making them abundantly capable of resisting the effects of acids, (provided the proper prophylactic and hygienic means are used) and endowing them with a lifelong exemption from decay, thereby enabling them to fulfill to the end of life the purpose of their creation. Myriads of teeth perish, mainly for the want of these elements, as other myriads fail, even after the expenditure of much skill and labor on them, for the same reason. These restorative elements may be found in bread made of unbolted wheat flour, oat meal, wheaten grits, beans, peas, beef and mutton. The Scotch are famous in history for their muscular and intellectual strength, which is ascribed mainly to their national diet, of which oat meal is aleading item. Dentists of experience know that the Scotch are famous for the strength and excellence of their teeth, which rarely, if ever, require their professional services, especially while they adhere to their national diet. It is only after emigration to, and settlement in this country, the habits and customs of which they generally adopt, that their teeth *fail*, chiefly because the elements which made their teeth so strong and healthy in Bonnie Scotland are wanting in our national diet.

The inroads made by caries of the teeth may to a great extent be arrested by filing and polishing when superficial, and by plugging with gold, or some other suitable material when it is deep seated. The material most generally employed and most highly endorsed, and deservedly too, is gold; chiefly for its ability to resist the action of acids, and the friction of mastication; it is also free from any deleterious agency—hence its presence in the mouth in large or small quantities is unobjectionable. It fills the bill better than any other material in the hands of an expert operator. Nevertheless it fails in conserving the teeth; sorry are we to say, it fails too frequently, even in the hands of the most skilful and conscientious. *Fails*, not because of any special defects in the manipulations of the operator, but because of the perishable nature of the material on which the operation is performed. So frail in many cases are these modern teeth—teeth who are starved to death—that the splendid golden creations, on which the devoted operator spent so much time, and concerning which he felt so much commendable pride, tumble into ruins, because of the perishable structure on which the gold was engrafted.

This predisposition to decay under the operation of the biological law of Heredity, which renders so many dental operations comparatively temporary, being conceded, the bad teeth of parents being transmitted to their offspring, in connection with our vicious system of living and the unscientific manner in which our foods are prepared, whereby all need of mastication is to a great extent superseded, together unite in demanding that there should be a reform in our dietectic habits, else the coming man will be practically worthless and a failure, as to all those ennobling physical and mental qualities, which unite in constituting the well developed and healthy specimen of the germs homo. Therefore, one of the conditions, without which no one can have good health as well as good teeth, is that this heredity to diseased conditions, physical as well as dental, must be as much as possible, thwarted by the use of all the available means possible for this purpose. Hence the dentist should be competent to advise his patients how to live, how to care for his physical, as well as his dental structure, in order to escape the doom resulting from negligence and inattention. Additional to the *best* professional service which he can possibly render, should be superadded the best digested advice and instruction in regard to habits of living, the foods which they eat, their preparation, personal cleanliness, including of course, most imperatively the teeth. Then we may reasonably hope, if such advice is heeded as it should be, our patrons will retain their teeth for a greater length of time, and certainly realize to a greater extent the utility and potentiality of Dental Surgery.

